# Prevention of Anterior Cruciate Ligament Injuries in Competitive Adolescent Alpine Skiers

**DOI:** 10.3389/fspor.2020.00011

**Published:** 2020-03-06

**Authors:** Maria Westin, Marita Löfgren Harringe, Björn Engström, Marie Alricsson, Suzanne Werner

**Affiliations:** ^1^Department of Molecular Medicine and Surgery, Karolinska Institutet, Stockholm Sports Trauma Research Center, Stockholm, Sweden; ^2^Department of Sports Science, Linnaeus University, Kalmar, Sweden

**Keywords:** alpine skiing, adolescent, ACL injury, knee injuries, awareness program, risk factor

## Abstract

Anterior cruciate ligament (ACL) injury is one of the most serious injuries among Swedish alpine ski high school students. An ACL injury forces the skier to stop skiing for several months, and some skiers even have to give up their skiing career. Therefore, an ACL injury prevention program might play an important role for alpine skiers. In the present study ski high school students have been followed in terms of ACL injuries during 1–2 ski seasons between 2006/2007 and 2012/2013. Alpine skiers studying at the Swedish ski high schools during the ski seasons 2011/2012 and 2012/2013 received a specific ACL injury prevention program (*n* = 305), while alpine skiers who attended a Swedish ski high school between the ski seasons 2006/2007 and 2010/2011 served as controls (*n* = 431). The prevention program was based on earlier studies and included indoor and outdoor exercises on snow focusing on core stability and neuromuscular control. Alpine skiing is an equilateral sport. Therefore, the goal of the prevention was to encourage the skiers to practice these exercises in order to perform equally good on both legs. The outcome measure consisted of the number and incidence of ACL injuries. The 2 years of prevention resulted in 12 ACL injuries (3.9%) compared with 35 ACL injuries during the control period (8.1%). The absolute risk rate showed a decreased incidence rate of −0.216 [CI −0.001–(−0.432)]/100 months attending a ski high school in favor of the intervention group. A prevention program focusing on the skier's ability to perform neuromuscular exercises equally good on both legs led to a reduction of ACL injuries.

## Introduction

Worldwide alpine skiing is a very popular winter sport (Hunter, 1999). It attracts both genders and different ages, and skiing performance depends on age and skiing level. Like other sports alpine skiing can, however, lead to severe injuries irrespective of skiing level (Westin et al., 2012; Bere et al., 2014; Stenroos and Handolin, 2014).

Several epidemiological studies in competitive alpine skiers have reported the knee to be the most frequently injured body part and anterior cruciate ligament (ACL) injury the most common diagnosis (Florenes et al., 2009; Bere et al., 2014; Stenroos and Handolin, 2014). From a 25-year follow-up of the French national ski team, Pujol et al. (2007) reported an incidence rate of 8.5 ACL injuries/100 ski seasons. An ACL injury is serious, and irrespective of gender it constitutes a risk for the skier's career as well as for early osteoarthritis (Lohmander et al., 2004; Nordenvall et al., 2014). These facts highlight the importance of ACL injury prevention.

In the epidemiological literature, van Mechelen's four steps model for developing a sport specific prevention program has been suggested (van Mechelen et al., 1992). The first step evaluates injury incidence and injury severity, the second step identifies injury mechanism and intrinsic as well as extrinsic injury risk factors, the third step consists of an intervention in terms of injury prevention based on steps one and two, and the fourth step evaluates the prevention strategies by repeating step one.

A number of both intrinsic and extrinsic risk factors for ACL injuries have been proposed (Smith et al., 2012a,b). However, to the best of our knowledge, only a few of these risk factors are sport specific for alpine skiing at the elite level. Raschner et al. (2012) found that impaired core strength was a critical factor for sustaining an ACL injury in young competitive ski racers. Westin et al. (2018) reported a higher risk to sustain an ACL injury in the left compared to the right knee. Bere et al. (2011a,b); Bere et al. (2013) studied the injury mechanism in World Cup skiers. From their video analysis of 20 ACL injuries they reported “slip and catch” to be the most common ACL injury mechanism (Bere et al., 2011a). The “slip and catch” mechanism means that the skier loose the pressure on the outer ski, while the inside edge of the outer ski is catching the snow and forces the knee into valgus and internal rotation. Moreover, Bere et al. (2011b) found that the majority of knee injuries occurred while skiing, and a technical as well as an inappropriate tactical mistake could lead to an ACL injury.

Still knowledge is sparse about alpine injury prevention, especially when it comes to adolescents. Changing the ski regulations by increasing the side cut radius and the ski length, Haaland et al. (2016) recently reported a reduction in the total injury incidence among skiers at the World Cup level. However, the side cut radius and the ski length change did not specifically influence the incidence of knee injuries (Haaland et al., 2016). For recreational skiers, there are two studies focusing on injury prevention by introducing a skiing education program (Ettlinger et al., 1995; Jlrgensen et al., 1998). Ettlinger et al. (1995) reported a reduction of 62% of serious knee sprains in skiers who had completed their “ACL-Awareness Program.” Jlrgensen et al. (1998) reported a decreased injury risk by showing an instructional ski video.

In Sweden, about 700 skiers hold a FIS-license, and nearly 100% of these alpine skiers, aged 16–20 years, are studying at a Swedish ski high school. Approximately, 60 new alpine skiers are entering the ski high schools every year. The skiers can study 3–4 years at a Swedish ski high school. The admission to a Swedish ski high school is based on points from the ranking system of the International Ski Federation (FIS). Entering one of these ski high schools may lead to membership of the national team and thereby a possibility to reach the world class in alpine skiing.

Hewett et al. (2010) have reported that a side-to-side difference could increase the injury risk in either the dominant or the non-dominant leg. The individual skier might rely too much on his (or her) dominant leg, which may result in too high a load of the knee joint. Contrarily, less muscle strength, and impaired coordination of the non-dominant leg when compared with the dominant leg could also be an injury risk factor (Hewett et al., 2010). Alpine skiing is an equilateral sport where the skier needs to perform equally well when performing ski turns to the left as well as to the right. There are heavy physical demands in competitive alpine skiers in terms of rapid speeds and high external loads (Berg et al., 1995; Hintermeister et al., 1995). To meet these demands it is likely that the skier needs very good bilateral leg muscle strength symmetry (Neumayr et al., 2003). However, physical conditioning also includes other physical variables such as core stability, balance, and coordination (Hydren et al., 2013).

Since the season 2006/2007, Swedish ski high school students, age 16–20 years, have been prospectively followed with respect to physical performance and injuries. Risk profiles have been reported and risk factors concluded (Westin et al., 2018). The present study is the third step in the model by van Mechelen et al. (1992) where an ACL injury prevention program based on previous findings is implemented. The aim of the present investigation was to evaluate whether a sports specific prevention program including different exercises could reduce the incidence of ACL injuries in competitive adolescent alpine skiers.

## Materials and Methods

### Study Design and Subjects

The present investigation is an ACL injury prevention study performed during a period of 21 months including two groups, an intervention group and a control group. The intervention group consisted of alpine skiers studying at a Swedish ski high school during the ski seasons 2011/2012 and 2012/2013. The control group consisted of 456 alpine skiers, who attended a ski high school between the seasons 2006/2007 and 2010/2011. Two ski seasons or 21 months attending a ski high school were the maximal time of exposure for a skier included in the present investigation. During this period the new changes of ski regulations by FIS were not yet implemented at the national level (Haaland et al., 2016). The reasons for using historical controls were 2-fold. Firstly, there would be a risk for crossover effects when some skiers performed a prevention program and other skiers did not. Secondly, in order to reduce the risk of a crossover effect a cluster randomization design would be appropriate, but the number of skiers would be a limited factor.

**Figure 1:**
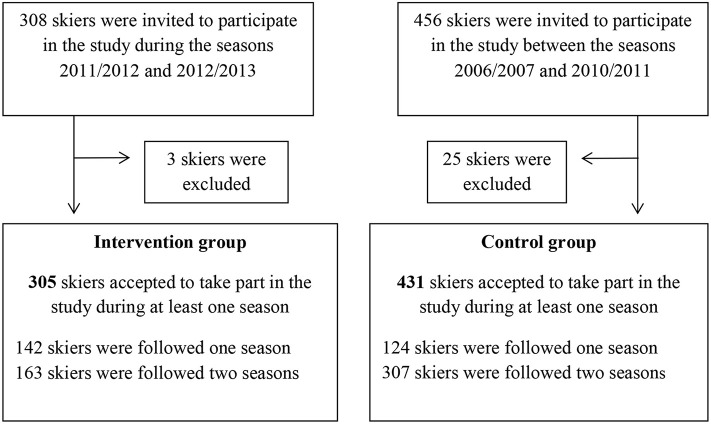
Flowchart of the alpine skiers throughout the study period.

A total of 28 skiers were excluded from the study for two reasons: either they did not complete their studies at the school (*n* = 21) or they left the school due to earlier injuries (*n* = 7) (Figure 1).

At baseline the alpine skiers answered a questionnaire including questions such as how many years they had been skiing, at what age they started to participate in ski competitions, how important they found alpine skiing, and whether the skier had had any skiing related injuries that stopped him or her from participating in alpine skiing. The FIS ranking was obtained from the link of FIS, and the skier's FIS ranking when entering the study was used. Demographic data are presented in Table 1.

**Table 1:** Specific characteristics of the alpine skiers at baseline.

	**Intervention group** **(*n* = 305)**	**Control group** **(*n* = 431)**
**Gender**	**Males (*n* = 148)** **Females (*n* = 157)**	**Males (*n* = 215)** **Females (*n* = 216)**
Age (year)^a^	17.1 ± 1.14	17.5 ± 1.23
Skiing experience (year)^a^	11.2 ± 2.31	11.3 ± 2.31
Competitive experience (year)^a^	8.6 ± 2.37	8.9 ± 2.29
FIS-rank SL (rank place)^b^	1,500 (27–5,261)	1,820 (14–5,895)
FIS-rank GS (rank place)^b^	1,831 (33–5,726)	2,102 (139–5,942)
Previous lower extremity injury	129 (42)	184 (43)
Previous ACL reconstructions^c^	23 (8) Males = 4 Females = 18	33 (8) Males = 4 Females = 27

*There were no significant differences between the groups*.

a *Mean and standard deviation*.

b *Median and range*.

c *Number and percentage*.

*SL, slalom; GS, giant slalom*.

### Intervention

#### ACL Injury Prevention Video

In collaboration with representatives from the Swedish Ski Federation and inspired by the Vermont Skiing Safety Research Group, an educational ACL injury prevention video was developed. The video was based on the injury profile (Westin et al., 2012) that identified intrinsic risk factors for ACL injuries in competitive adolescent alpine skiers (Westin et al., 2018) and their experience of having a safer/better ski turn to the right or to the left. The video was produced by two professional film producers.

The video (*https://www.youtube.com/watch?v=l-9CrG7lmAg&t=10s**)* included information about ACL injuries in competitive alpine skiers and how to possibly avoid ACL injury situations while skiing. Additionally, the video consisted of three indoor and three outdoor exercises on snow focusing on core stability and neuromuscular control (Hewett et al., 2010). The Indoor exercises consisted of the one leg hop test for distance (Itoh et al., 1998), the square hop test (Itoh et al., 1998), and the single leg squat (Hydren et al., 2013). The outdoor exercises that were suggested by the Swedish Ski Federation consisted of the shuffle, the back and forth, and turns with lifted inner ski. The skiers were instructed to be aware of whether they were able to perform the exercises equally well on both legs and if they could perform equally well ski turns to the left as to the right.

#### First Year of Prevention

The prevention program was introduced in September 2011, and all ski coaches were taught how to implement the preventive strategies. The video was used as a support in order to educate the ski students about ACL injury prevention. They were instructed to watch the video every third week between September and November (preseason) and once each month between December and April (competition season). The education included information about identified risk factors for ACL injuries in alpine skiing and the importance of stimulating the skiers to regularly perform the suggested exercises. The coaches were informed that the ACL of the left leg was more often injured than that of the right leg. Therefore, the guidelines were to perform the exercises equally well on both legs.

#### Second Year of Prevention

Prior to the second year the test leader (MW) paid a visit at each ski high school in order to educate the new ski students and also to remind the other ski students as well as their coaches about the prevention program.

During the entire study period the test leader had monthly contact with the ski students as well their coaches. The reason for these contacts was to make sure that all ACL injuries were collected and to be informed about how the skiers complied with the suggested exercises. Their compliance with the training was collected using a questionnaire. At the end of the second year all coaches were reinvited to a meeting in order to secure that all ACL injuries had been recorded and reported to the main test leader.

### Injury Report

All reported ACL injuries in the present study were total ACL ruptures, diagnosed by experienced orthopedic surgeons, and confirmed with MRI and/or arthroscopy.

Every month the test leader was in contact with the skiers by email and with the coaches by telephone asking about possible reinjuries or new ACL injuries. In case of an ACL injury the skier filled out a standardized injury form. Besides the date of injury and the injury mechanism, this form included questions about ski discipline, weather condition, visibility, slope conditions as well as temperature (Celsius degree) when the ACL injury occurred. In addition, this form included a question about physical fatigue and history of possible earlier injuries.

### Statistical Analysis

Data is presented according to guidelines for reporting observational studies (Rothman, 2002). Descriptive statistics is presented for categorical data with median and range and for continuous data with mean and standard deviation. ACL injury prevalence was calculated as the number of ACL injuries divided by the total number of skiers, and a Chi-2 test was used to determine whether there was a significant difference with respect to ACL injuries between the groups. Time of exposure was calculated as the total number of months that a skier was attending a ski high school, and the ACL injury incidence (IR) was reported as the total number of ACL injuries per 100 months that a skier was attending a ski high school. A 95% confidence interval was estimated. The absolute rate reduction of the intervention was calculated as absolute incidence rate differences [IRD = IR (intervention group) – IR (control group)] with 95% confidence interval (Rothman, 2002). The time to the occurrence of an ACL injury between the groups was calculated and presented with the Kaplan–Meier survival curve. All *P*-values were two-tailed with *p* < 0.05 considered statistically significant. Data were analyzed using Statistica 12, StatSoft®, Inc. Tulsa OK, USA.

## Results

During the intervention seasons 12 skiers (5 males, 7 females) sustained 12 complete ACL injuries. This resulted in a prevalence of 3.9% and an injury incidence of 0.26/100 months (95% CI 0.11–0.41) for a skier attending a ski high school. Eight of the injuries were first time ACL injuries, and two were re-injuries (Table 2).

**Table 2:** Side distribution of ACL injuries during the study period.

	**Intervention group**		**Control group**	
	**Males** **(*n* = 5)**	**Females** **(*n* = 7)**	**Males** **(*n* = 14)**	**Females** **(*n* = 21)**
First time ACL injury (*n*, side)	4 (2 left, 2 right)	4 (2 left, 2 right)	11 (8 left, 3 right)	13 (9 left, 4 right)
ACL re-injury (*n*, side)	1 (left)	1 (left)	2 (left)	5 (4 left, 1 right)
ACL injury of the contralateral knee (*n*)		2	1	2
Third time ACL injury (*n*)				1

During the control seasons there were 33 skiers (12 males, 21 females) that sustained a total of 35 complete ACL ruptures resulting in a prevalence of 8.1% and an injury incidence of 0.48/100 months (95% CI 0.32–0.64) for a skier attending a ski high school. Twenty-four of the ACL injuries were first time ACL injuries and seven were re-injuries (Table 2).

**Figure 2:**
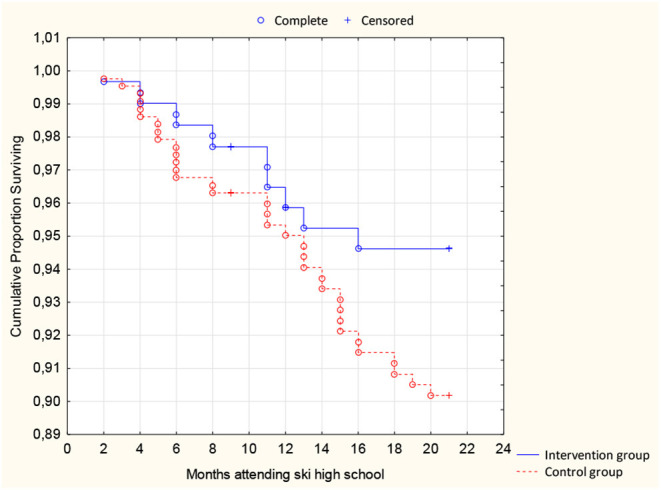
Kaplan–Meier survival curve for ACL injuries in the intervention group and the control group.

The absolute risk rate showed a decreased incidence rate of −0.216 (CI −0.001 to (−0.432)/100 months for a skier attending a ski high school in favor of the intervention group. A Hazard ratio of 0.56 (CI 0.29–1.08) was shown and reported according to the Kaplan–Meier survival curve (Figure 2). The number of ACL injuries in the intervention group was significantly reduced (*p* = 0.03).

### Compliance With the Prevention Program

The ACL injury prevention program was included in the skiers' ordinary training. Out of the 305 skiers 94 (42%) answered the questionnaire about their compliance, and 75% reported that they had watched the video 1–5 times, 62% that their training had been focused on equilateral skiing, and 41% of the skiers had used the suggested exercises to find out whether their performance was equally well on both legs.

## Discussion

The overall aim of this investigation was to study whether a specific prevention program could reduce or prevent the number of ACL injuries in competitive adolescent alpine skiers.

The result showed a reduction of the incidence rate with −0.216 ACL injuries/100 months for a skier attending a ski high school. This means a reduction of 45% during the prevention period. Solely a few studies on ACL injury prevention in alpine skiing exist. In contrast to the present study Haaland et al. (2016) tried to prevent injuries by equipment changing focusing on intrinsic risk factors. To the best of our knowledge the present investigation is, however, the first one in adolescent alpine skiers at competitive level. Ettlinger et al. (1995) reported a decreased ACL injury rate as a result of a special educational program for ski instructors and ski patrollers in order to avoid ACL injury risk situations and also how to fall when off balance and how to stop after a fall. Like Ettlinger et al. (1995) the authors of the present study have tried to teach both the ski coaches and the alpine skiers to be aware of possible ACL injury risk situations. Awareness of certain risk situations may help to reduce the skier's technical mistake and thereby preventing an ACL injury (Bere et al., 2011b).

In a study of ACL injury risk factors we found that ACL injured alpine skiers showed an increased side-to-side difference when performing the one leg hop test for distance compared to the uninjured skiers. In the same investigation we also found that the skier's left knee was more injured than their right knee, which might at least to some extent, be due to this side-to-side difference (Westin et al., 2018). A recent publication about normative data when performing hop tests showed various results in terms of the best leg (left or right) for hop performance (Hildebrandt et al., 2015). When it comes to another hop test, the one leg counter movement jump, hop performance of the dominant leg was superior to the non-dominant leg (Hildebrandt et al., 2015). Alpine skiing is a multifactorial sport including a number of different physiological demands on the skier (Hydren et al., 2013). Furthermore, alpine skiing is an equilateral sport, meaning that the same physical demands are put on both legs. Therefore, the goal of the prevention program in the present investigation was to perform different types of exercises, indoors as well as outdoors on snow, with focus on how to perform equally well on the left and right legs. In female athletes it has been reported that those with a side-to-side asymmetry in terms of muscle strength are at a greater risk of an ACL injury than athletes without this asymmetry (Hewett et al., 2005) Whether this goes for male athletes, as well, is not known.

Moreover, Hewett et al. (2010) have pointed out the importance of a good neuromuscular balance between the quadriceps and hamstring muscles and between the dominant and non-dominant leg as well as a good postural control in order to prevent ACL injuries. These aspects may be of potential interest especially in an equilateral sport like alpine skiing. Therefore, in the present study, the prevention program was focused on each skier's awareness of his (or her) side-to-side ability when performing the suggested exercises, indoors as well as outdoors on snow. Thus, the goal was to perform equally well on both legs. Majority of earlier publications on ACL injury prevention are in team ball sports, such as soccer (Mandelbaum et al., 2005; Walden et al., 2012) and handball (Myklebust et al., 2003). These studies have been focused on neuromuscular warm-up programs which have led to a reduction of ACL injuries (Myklebust et al., 2003; Mandelbaum et al., 2005; Walden et al., 2012). Similar to these team ball studies we found that a training concept focusing on neuromuscular exercises can reduce the ACL incidence rate. As suggested by Finch (2006), the next step following this investigation should be to study motivators and barriers for the implementation of a prevention program. However, more studies specifically tailored for reducing ACL injuries in competitive adolescent alpine skiers are needed.

### Strengths

A strength when using a prospective cohort design is that you are able to calculate the absolute risk. The absolute risk associated with exposure is of greater interest than the relative risk due to the statistical method. Another strength is that the preventive strategies implemented in young athletes may be more successful if both the skiers and their coaches are fully informed. In the present study, the coaches and the skiers were instructed to work together using an education program in order to reduce or prevent ACL injuries in competitive adolescent alpine skiers including exercises, both indoors and outdoors on snow, which also could be considered as strength of the study.

### Limitations

Using historical controls may be seen as a study limitation, but the reasons were 2-fold. Firstly, the alpine ski students are regularly meeting each other at both national and international ski camps and ski competitions. Consequently, it was impossible to randomize some of the skiers to an intervention group and others to a control group without the risk of a crossover effect. Secondly, due to the limited number of alpine ski students in Sweden the use of historic controls was the way to being able to perform a prevention study of this type. More alpine ski students and/or a higher number of studied ski seasons might have been more appropriate. However, the present investigation was a cohort consisting of all Swedish alpine ski students with more than 200 students per year, and they are solely studying 3 or 4 years at a Swedish ski high school. Therefore, there would be huge logistical problems to increase the number of skiers and/or ski seasons.

Only 42% of the students in the intervention group replied on the compliance form, and it is therefore not trustworthy. Finch et al. (2011) concluded that a prevention program must be carried out regularly and be integrated with the normal sport specific training. In the present study both coaches and skiers were educated in the program, and the exercises were initially performed together with the coaches. Maybe that was the reason that the number of ACL injuries was reduced. From a psychological point of view, the close contact throughout the entire study period between the main test leader and the coaches and their alpine ski students could also have been the reason for the reduction of ACL injuries.

## Conclusion

A prevention program of ACL injuries led to a 45% reduction of ACL injuries in Swedish alpine ski high school students. This indicates that an ACL injury prevention program consisting of neuromuscular exercises both indoors and outdoors on snow can prevent ACL injuries in competitive adolescent alpine skiers.

## Data Availability Statement

The datasets generated for this study are available on request to the corresponding author.

## Ethics Statement

The present investigation was approved by the Regional Ethics Committee in Stockholm, Dnr 2006/833-31/1. Prior to entering the study all skiers were given both oral and written information. Participation was voluntary, and all skiers consented to participate.

## Author Contributions

MW conducted all data collecting. MW and MH conducted data analysis and graphical representation of the findings. All authors contributed to conception and design of the work, drafted it, revised it critically for important intellectual content, approved the final version of the manuscript, and agreed to be accountable for all aspects of the work in ensuring that questions related to the accuracy or integrity of any part of the work are appropriately investigated and resolved. All persons designated as authors qualify for authorship, and all those who qualify for authorship are listed.

### Conflict of Interest

The authors declare that the research was conducted in the absence of any commercial or financial relationships that could be construed as a potential conflict of interest.

## References

[B1] Bere T. Florenes T. W. Krosshaug T. Koga H. Nordsletten L. Irving C.. (2011a). Mechanisms of anterior cruciate ligament injury in World Cup alpine skiing: a systematic video analysis of 20 cases. Am. J. Sports Med. 39, 1421–1429. 10.1177/0363546511405147 21515807

[B2] Bere T. Florenes T. W. Krosshaug T. Nordsletten L. Bahr R. (2011b). Events leading to anterior cruciate ligament injury in World Cup Alpine Skiing: a systematic video analysis of 20 cases. Br. J. Sports Med. 45, 1294–1302. 10.1136/bjsports-2011-090517 22067283

[B3] Bere T. Florenes T. W. Nordsletten L. Bahr R. (2014). Sex differences in the risk of injury in World Cup alpine skiers: a 6-year cohort study. Br. J. Sports Med. 48, 36–40. 10.1136/bjsports-2013-092206 23673520

[B4] Bere T. Mok K. M. Koga H. Krosshaug T. Nordsletten L. Bahr R. (2013). Kinematics of anterior cruciate ligament ruptures in World Cup alpine skiing: 2 case reports of the slip-catch mechanism. Am. J. Sports Med. 41, 1067–1073. 10.1177/0363546513479341 23449837

[B5] Berg H. E. Eiken O. Tesch P. A. (1995). Involvement of eccentric muscle actions in giant slalom racing. Med. Sci. Sports Exerc. 27, 1666–1670. 10.1249/00005768-199512000-00013 8614323

[B6] Ettlinger C. F. Johnson R. J. Shealy J. E. (1995). A method to help reduce the risk of serious knee sprains incurred in alpine skiing. Am. J. Sports Med. 23, 531–537. 10.1177/036354659502300503 8526266

[B7] Finch C. (2006). A new framework for research leading to sports injury prevention. J. Sci. Med. Sport. 9:3–9; discussion 10. 10.1016/j.jsams.2006.02.009 16616614

[B8] Finch C. F. White P. Twomey D. Ullah S. (2011). Implementing an exercise-training programme to prevent lower-limb injuries: considerations for the development of a randomised controlled trial intervention delivery plan. Br. J. Sports Med. 45, 791–796. 10.1136/bjsm.2010.081406 21393258

[B9] Florenes T. W. Bere T. Nordsletten L. Heir S. Bahr R. (2009). Injuries among male and female World Cup alpine skiers. Br. J. Sports Med. 43, 973–978. 10.1136/bjsm.2009.068759 19945979

[B10] Haaland B. Steenstrup S. E. Bere T. Bahr R. Nordsletten L. (2016). Injury rate and injury patterns in FIS World Cup Alpine skiing (2006–2015): have the new ski regulations made an impact? Br. J. Sports Med. 50, 32–36. 10.1136/bjsports-2015-095467 26559877

[B11] Hewett T. E. Myer G. D. Ford K. R. Heidt R. S. Jr Colosimo A. J. McLean S. G.. (2005). Biomechanical measures of neuromuscular control and valgus loading of the knee predict anterior cruciate ligament injury risk in female athletes: a prospective study. Am. J. Sports Med. 33, 492–501. 10.1177/0363546504269591 15722287

[B12] Hewett T. E. Ford K. R. Hoogenboom B. J. Myer G. D. (2010). Understanding and preventing acl injuries: current biomechanical and epidemiologic considerations - update 2010. N. Am. J. Sports Phys. Ther. 5, 234–251. 21655382 PMC3096145

[B13] Hildebrandt C. Muller L. Zisch B. Huber R. Fink C. Raschner C. (2015). Functional assessments for decision-making regarding return to sports following ACL reconstruction. Part I: development of a new test battery. Knee Surg. Sports Traumatol. Arthrosc. 23, 1273–1281. 10.1007/s00167-015-3560-5 25682164 PMC4555192

[B14] Hintermeister R. A. O'Connor D. D. Dillman C. J. Suplizio C. L. Lange G. W. Steadman J. R. (1995). Muscle activity in slalom and giant slalom skiing. Med. Sci. Sports Exerc. 27, 315–322. 10.1249/00005768-199503000-00005 7752856

[B15] Hunter R. E. (1999). Skiing injuries. Am. J. Sports Med. 27, 381–389. 10.1177/03635465990270032101 10352778

[B16] Hydren J. R. Volek J. S. Maresh C. M. Comstock B. A. Kraemer W. J. (2013). Review of strength and conditioning for alpine ski racing. Strength Cond. J. 35, 10–28. 10.1519/SSC.0b013e31828238be 23207888

[B17] Itoh H. Kurosaka M. Yoshiya S. Ichihashi N. Mizuno K. (1998). Evaluation of functional deficits determined by four different hop tests in patients with anterior cruciate ligament deficiency. Knee. Surg. Sports Traumatol. Arthrosc. 6, 241–245. 10.1007/s001670050106 9826806

[B18] Jlrgensen U. Fredensborg T. Haraszuk J. P. Crone K. L. (1998). Reduction of injuries in downhill skiing by use of an instructional ski-video: a prospective randomised intervention study. Knee. Surg. Sports Traumatol. Arthrosc. 6, 194–200. 10.1007/s001670050098 9704328

[B19] Lohmander L. S. Ostenberg A. Englund M. Roos H. (2004). High prevalence of knee osteoarthritis, pain, and functional limitations in female soccer players twelve years after anterior cruciate ligament injury. Arthritis Rheum. 50, 3145–3152. 10.1002/art.20589 15476248

[B20] Mandelbaum B. R. Silvers H. J. Watanabe D. S. Knarr J. F. Thomas S. D. Griffin L. Y.. (2005). Effectiveness of a neuromuscular and proprioceptive training program in preventing anterior cruciate ligament injuries in female athletes: 2-year follow-up. Am. J. Sports Med. 33, 1003–1010. 10.1177/0363546504272261 15888716

[B21] Myklebust G. Engebretsen L. Braekken I. H. Skjolberg A. Olsen O. E. Bahr R. (2003). Prevention of anterior cruciate ligament injuries in female team handball players: a prospective intervention study over three seasons. Clin. J. Sport Med. 13, 71–78. 10.1097/00042752-200303000-00002 12629423

[B22] Neumayr G. Hoertnagl H. Pfister R. Koller A. Eibl G. Raas E. (2003). Physical and physiological factors associated with success in professional alpine skiing. Int. J. Sports Med. 24, 571–575. 10.1055/s-2003-43270 14598192

[B23] Nordenvall R. Bahmanyar S. Adami J. Mattila V. M. Fellander-Tsai L. (2014). Cruciate ligament reconstruction and risk of knee osteoarthritis: the association between cruciate ligament injury and post-traumatic osteoarthritis. a population based nationwide study in Sweden, 1987-2009. PLoS ONE. 9:e104681. 10.1371/journal.pone.0104681 25148530 PMC4141753

[B24] Pujol N. Blanchi M. P. Chambat P. (2007). The incidence of anterior cruciate ligament injuries among competitive Alpine skiers: a 25-year investigation. Am. J. Sports Med. 35, 1070–1074. 10.1177/0363546507301083 17468379

[B25] Raschner C. Platzer H. P. Patterson C. Werner I. Huber R. Hildebrandt C. (2012). The relationship between ACL injuries and physical fitness in young competitive ski racers: a 10-year longitudinal study. Br. J. Sports Med. 46, 1065–1071. 10.1136/bjsports-2012-091050 22968156

[B26] Rothman J. K. (2002). Epidemiology- An Introduction. New York, NY: Oxford.

[B27] Smith H. C. Vacek P. Johnson R. J. Slauterbeck J. R. Hashemi J. Shultz S.. (2012a). Risk factors for anterior cruciate ligament injury: a review of the literature - part 1: neuromuscular and anatomic risk. Sports Health. 4, 69–78. 10.1177/1941738111428281 23016072 PMC3435896

[B28] Smith H. C. Vacek P. Johnson R. J. Slauterbeck J. R. Hashemi J. Shultz S.. (2012b). Risk factors for anterior cruciate ligament injury: a review of the literature-part 2: hormonal, genetic, cognitive function, previous injury, and extrinsic risk factors. Sports Health. 4, 155–161. 10.1177/1941738111428282 23016083 PMC3435909

[B29] Stenroos A. J. Handolin L. E. (2014). Alpine skiing injuries in Finland - a two-year retrospective study based on a questionnaire among Ski racers. BMC Sports Sci. Med. Rehabil. 6:9. 10.1186/2052-1847-6-9 24565467 PMC3974042

[B30] van Mechelen W. Hlobil H. Kemper H. C. (1992). Incidence, severity, aetiology and prevention of sports injuries. A review of concepts. Sports Med. 14, 82–99. 10.2165/00007256-199214020-00002 1509229

[B31] Walden M. Atroshi I. Magnusson H. Wagner P. Hagglund M. (2012). Prevention of acute knee injuries in adolescent female football players: cluster randomised controlled trial. BMJ. 344:e3042. 10.1136/bmj.e3042 22556050 PMC3342926

[B32] Westin M. Alricsson M. Werner S. (2012). Injury profile of competitive alpine skiers: a five-year cohort study. Knee. Surg. Sports Traumatol. Arthrosc. 20, 1175–1181. 10.1007/s00167-012-1921-x 22349602

[B33] Westin M. Harringe M. L. Engstrom B. Alricsson M. Werner S. (2018). Risk Factors for anterior cruciate ligament injury in competitive adolescent alpine skiers. Orthop. J. Sports Med. 6:2325967118766830. 10.1177/2325967118766830 29780835 PMC5954346

